# How to Understand Behavioral Patterns in Big Data: The Case of Human Collective Memory

**DOI:** 10.3390/bs9040040

**Published:** 2019-04-16

**Authors:** Steven A. Frank

**Affiliations:** Department of Ecology and Evolutionary Biology, University of California, Irvine, CA 92697-2525, USA; safrank@uci.edu; Tel.: +1-949-824-2244

**Keywords:** collective behavior, cultural transmission, signal frequency analysis

## Abstract

Simple patterns often arise from complex systems. For example, human perception of similarity decays exponentially with perceptual distance. The ranking of word usage versus the frequency at which the words are used has a log-log slope of minus one. Recent advances in big data provide an opportunity to characterize the commonly observed patterns of behavior. Those observed regularities set the challenge of understanding the mechanistic processes that generate common behaviors. This article illustrates the problem with the recent big data analysis of collective memory. Collective memory follows a simple biexponential pattern of decay over time. An initial rapid decay is followed by a slower, longer lasting decay. Candia et al. successfully fit a two stage model of mechanistic process to that pattern. Although that fit is useful, this article emphasizes the need, in big data analyses, to consider a broad set of alternative causal explanations. In this case, the method of signal frequency analysis yields several simple alternative models that generate exactly the same observed pattern of collective memory decay. This article concludes that the full potential of big data analyses in the behavioral sciences will require better methods for developing alternative, empirically testable causal models.

## 1. Introduction

Complex processes often express a simple pattern. For example, city size [1], corporation size [2], and word usage [3] follow Zipf’s law, in which a log-log plot for the rank of size versus size has a slope of minus one.

Simple patterns also arise in human category generalization [4], in the abundance of different species of ecological samples [5], and in the age of onset of the various causes of human mortality [6].

Solving the puzzle of simple regularity from diverse complexity is an essential challenge in understanding the relation between pattern and process.

The first step for any such puzzle is to see that a simple common pattern exists. The second step is to draw up a list of alternative explanations that can be tested.

A primary challenge recurs. Simple regularity often arises when diverse processes lead to the same pattern. One might say that a common pattern is common because it is the outcome of so many different underlying processes [7,8].

This likely relation between diverse processes and simple regularity must be kept in mind. There is a natural tendency to jump toward the first particular process that matches the regularity that we see.

Big data greatly enhances the opportunity to discover the simple regularities that characterize particular problems. So it is timely to consider how we may raise the standards by which we develop and evaluate hypotheses of mechanistic process.

I illustrate these issues with the recent big data characterization of a simple pattern in human collective memory [9]. The success of human group recall for past events decays with time. The decay follows a biexponential pattern. An initial exponential decay happens relatively quickly. A second slower decay happens relatively slowly.

The biexponential decay pattern characterizes the collective memory of songs, movies, biographies, and the citations to academic articles and patents. That simple regularity exists independently of any particular mechanistic explanation or interpretation.

The current biological literature follows a standard approach to puzzles of observed regularity. Propose a plausible mechanistic model. Show that the model generates the observed pattern. Note that alternatives are possible, but do not consider those alternatives. Let the fit of mechanism to pattern implicitly express tentative victory in the absence of good alternatives.

This approach is not incorrect. However, it is almost certainly wrong. Common patterns are common because the same simple pattern arises from a diverse set of underlying processes. We need to understand the generic aspect that unifies that diverse set of underlying processes. Such understanding is not easy to achieve. However, ignoring the real problem does not help.

I do not know exactly how this should be done. I do know that we need more discussion and thought devoted to these issues if we are to benefit fully from the modern opportunities of big data research. There are already several known approaches that can help.

In this article, I use the simple regularity of collective memory as an example. I begin with the mechanistic explanation presented by Candia et al. [9]. I then show a variety of alternative mechanisms consistent with the data.

This article is not a criticism of Candia et al. [9]. Their characterization of the simple regularity of collective memory by using a big data approach is a major contribution. Simple patterns set the puzzles that define disciplines.

Although I will show several alternative explanations that also match the data, in the end, the model by Candia et al. [9] remains as good as any alternative. But my conclusions differ in two ways.

First, I will provide several potentially testable alternative mechanistic models for the study of human collective memory. Such alternatives provide the essential next step to move this field ahead.

Second, I argue that the culture of big data analysis and interpretation needs to change. The success of finding a simple pattern should be followed by the demand for characterizing the set of alternative mechanisms and their generic properties. The fit of a single, particular mechanistic model typically misleads.

## 2. The Biexponential Pattern

**Figure 1:**
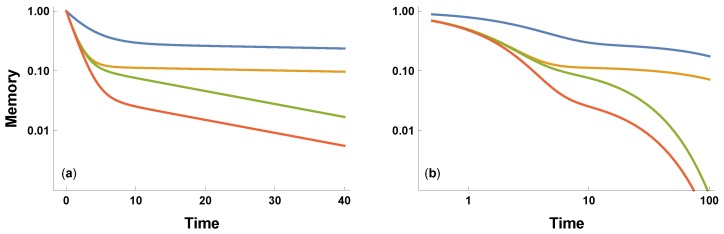
Biexponential decay of a signal in Equation (1). The same parameters are used for the plots on (**a**) semi-log axes and (**b**) log-log axes. The parameters for the curves from top to bottom are: (a,b,c)=(0.35,0.005,0.408),(0.85,0.005,0.134),(0.85,0.05,0.143),(0.78,0.05,0.043). The parameters match Figure 2 of Candia et al. [9], associated with their parameterization given in Equation (2) and the parameter equivalences: a=p+r; b=q; c=r/(p−q); and N=1. The inverse parameter equivalences are also useful: p=(a+bc)/(1+c); q=b; and r=c(a−b)/(1+c).

Let the intensity of collective memory be m(t) at time *t*. Set m(0)=1 as the memory intensity at the time of the initial event. A biexponential pattern arises when memory decays as the sum of a fast and a slow exponential process

(1) $$m\left(t\right)=\frac{e^{-at}+ce^{-bt}}{1+c},$$

in which *a* describes the initial fast decay, and *b* describes the subsequent slow decay, with a>b. The relative weighting of the processes is c≥0. Figure 1 illustrates the combination of fast and slow memory decay for various parameters.

Candia et al. show that biexponential decay arises for the collective memory of several different kinds of cultural phenomena. How can we explain that simple, widespread regularity of pattern?

## 3. Candia et al.’s Mechanistic Model

Candia et al. developed a specific mechanistic model of collective memory. Their model leads to the biexponential form of Equation (1). However, they interpret the parameters in terms of their particular mechanistic model. Their expression for the biexponential pattern is

(2) $$m\left(t\right)=\frac{N}{p+r-q}(\left(p-q\right)e^{-(p+r)t}+re^{-qt}),$$

in which they use S(t) instead of m(t) for the memory signal. Candia et al. obtain this expression by assuming that, after a particular event at t=0, collective memory decays by two distinct processes.

At first, decay happens relatively rapidly during a period dominated by short-lived communicative processes, such oral communication or simple messaging communication. The intensity of that first process of communication stimulates a second process of storing cultural memory in written or other relatively long-lasting forms. Cultural memory then decays over time, but at a slower rate than communicative memory.

**Figure 2:**
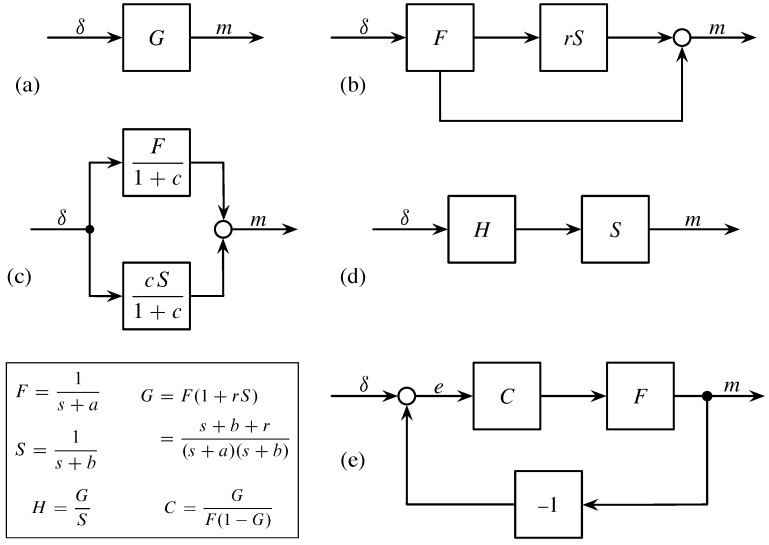
Alternative models that yield identical biexponential decay. The text describes each panel. The parameters can be matched to the generic model in Equation (1). with r=c(a−b)/(1+c).

The diagram in Figure 2b expresses the dynamics of their model. External stimulation of memory happens by an input signal, δ(t). We can often consider a strong input at the time of a particular event, with δ large at t=0 and zero after the initial input.

We then think of the initial event as filtered through a fast decay process, *F*, which by itself follows exponential decay, $e^{-at}$. The output from that fast decay contributes to the memory signal through two pathways.

The lower arrow in Figure 2b goes directly to the circle, which denotes the addition of inputs. The upper arrow feeds into a slow decay process, *S*, with exponential decay $e^{-bt}$. The slow process filters the output from the fast process, with a weighting of the fast output by the parameter, *r*. Adding the signal paths yields the biexponential pattern of memory in Equation (1).

## 4. Alternative Mechanistic Models

Candia et al. showed that the data form a pattern consistent with biexponential decay. Their particular mechanistic model in Figure 2b leads to the biexponential pattern.

That fit between observed pattern and explanatory causal process is useful. However, if we are to make sense of the patterns in big data, then we must start with the set of alternative causal models that fit the data. Typically, many alternative causal schemes fit a simple, widely observed pattern.

Once we have a clear set of alternatives, we can then consider how to test which alternative is most likely. We can also consider the extent to which various alternatives arise in different circumstances, all leading to the same observed pattern.

The block diagrams in Figure 2 provide a simple way in which to study alternative mechanistic models with the same biexponential dynamics of decay. Each diagram shows how to trace an input signal, δ, through a variety of processes that filter, transform, and combine the system’s internal signals to produce the final output signal, *m*.

My purpose is to show how easily one can generate alternative causal models. I do not argue in favor of any particular alternative. The data in Candia et al. do not discriminate between these alternatives. My point is that the first step in testing among alternatives is figuring out what those alternatives might be.

### 4.1. Structure of Alternative Process

Figure 2a shows the structure of all models. The input, δ, represents the external signals that stimulate the system’s memory. Such signals include the initial publication of an article, the filing a patent, or the initial release of a song. Any additional external processes that influence memory also enter through δ.

The block *G* describes all of the system’s internal processes. The system takes input, δ, and produces output memory, *m*. Any system that transforms inputs to outputs in the same way has the same memory dynamics. Thus, we can search for alternative models by considering the variety of processes that together have the same *G*.

In this case, we want the system, *G*, to match Candia et al.’s model in Figure 2b. The theory of signal processing provides a convenient way to express that model [10,11,12]. The dynamics of fast exponential decay, $e^{-at}$, has Laplace transform F=1/(s+a). The dynamics of slow exponential decay, $e^{-bt}$, has Laplace transform S=1/(s+b).

With Laplace transforms, we can follow a signal through a cascade of processes. For each separate process, we multiply the signal going into each process block by the Laplace transform of the process within that block. The output for that block may then be used as the input for another block.

In Figure 2b, the lower path is *F*. The upper direct path from input, δ, to output, *m*, is the product rFS. So the overall transformation is

G=F+rFS=F(1+rS).

To obtain equivalent system dynamics, we can search for alternative systems in which the overall internal processing equals *G*.

### 4.2. Additive Parallel Model

Figure 2c presents the simplest alternative model. The input stimulates two independent exponential decay processes, *F* and *S*. Those processes act in parallel. Their outputs combine additively to yield the final memory signal, which is equivalent to the memory output signal of the original model in Figure 2b. To match the parameters of the original model, we use the fact that

$$G=F\left(1+rS\right)=\frac{F+cS}{1+c},$$

in which the right side expresses the additive parallel model. It is easy to see that the inverse Laplace transform of the right side takes us back to the expression for the dynamics of collective memory in Equation (1).

The data in Candia et al. do not discriminate between the models in Figure 2b,c. The same final pattern of collective memory arises if, as in Figure 2b, the initial fast process of communicative memory directly influences the slower cultural memory process or if, as in Figure 2c, the slow and fast processes act independently.

Some studies from the prior literature on collective memory favor Candia et al.’s model over the additive parallel model [13,14,15,16]. However, the data do not discriminate between these alternative models. Other prior studies discuss various alternative processes [17]. Those earlier studies have typically not been developed into simple models that compare easily with the patterns in the big data analysis.

### 4.3. Multiplicative Series Model

In Figure 2d, the input is first transformed by a process, *H*. That initial transformed signal then passes through the slow exponential decay process, *S*. Because signals multiply, the overall system is G=HS. To match *G*, the first process must be H=G/S.

The following section on the frequency analysis of signals discusses how to interpret *H* and other components in these models. Before turning to interpretation, consider the final alternative explanation.

### 4.4. Exponential Decay with Feedback

The model in Figure 2e assumes that fast exponential decay, *F*, sets the baseline response. That fast decay is modulated by a feedback process. The final output from the fast decay, the memory signal, *m*, returns as an additional input into the system. The actual input at any time is the feedback difference between the external signal and the output, e(t)=δ(t)−m(t).

When the memory output signal is larger than the current input, then memory tends to decay. When the current input is larger than the current memory, then that stimulation tends to increase the memory signal. Such self-correcting feedback control is a natural aspect of many systems that respond to external inputs.

The component *C* represents the process by which the system filters the feedback input signal. For example, the system might tend to enhance certain types of feedback input. Such enhancement would increase the memory of events with certain characteristics, while perhaps ignoring events with other kinds of signal characteristics.

In this case, we can find the process, *C*, that makes the overall feedback process equivalent to Candia et al.’s model. The logic of block diagrams and Laplace transforms requires that *C* take on the value given in the box of Figure 2.

## 5. Frequency Interpretation of Process

The Laplace transform approach has two benefits. First, we can easily compare different causal schemes with the same overall dynamics. Second, we can interpret the various component causes within a model in terms of how they respond to different frequencies of inputs.

Engineers often use frequency response as a tool to analyze how a system works [10,11,12]. In this section, I provide a rough intuitive summary of the general principles. I then apply the method to the analysis of collective memory. The frequency approach provides an additional method for empirical tests that can discriminate between alternative models.

### 5.1. Exponential Decay as a Low Pass Filter

A component with exponential decay dynamics, $\lambda e^{-\lambda t}$, has Laplace transform L=λ/(s+λ). Roughly speaking, we can think of *s* as the frequency of fluctuations in the input signal to this process. The output is a signal with the same frequency of fluctuations, with the intensity of the signal multiplied by *L*.

The process *L* acts as a low pass filter on input signals. For low input frequency, s→0, the value of *L* is close to one. In other words, *L* passes through low frequency inputs with little change. For high input frequency, with large *s*, the value of *L* declines. In other words, *L* reduces or blocks high frequency inputs.

**Figure 3:**
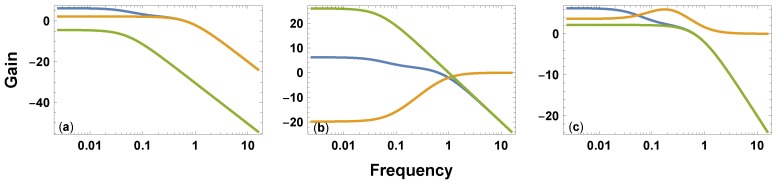
Frequency response of alternative models that yield identical biexponential decay. Each curve shows the relation between the frequency of input and the gain, which is the amount by which a process multiplies an input signal. The parameters match the red curve of Figure 1. The blue curve is the response of the total system, *G*, which is the same in all cases. The gain expresses the classic signal processing scale of $20\log_{10}\left(\mathrm{gain}\right)$. A value of zero corresponds to gain of one, which means that the process passes the signal without change. Values of less than zero reduce the signal intensity, and values above zero enhance the signal intensity. (**a**) The combination of two low pass filters in Candia et al.’s model of Figure 2b. A similar pattern describes the additive parallel model in Figure 2c. (**b**) The combination of high and low pass pass filters in series in Figure 2d. (**c**) A low pass filter in green combined in a self-correcting feedback loop with a filter that enhances intermediate resonant frequencies in gold.

The gold curve in Figure 3a illustrates the relatively fast exponential decay process, F=1/(s+a), as a low pass filter. The green curve shows the relatively slow process, rS=r/(s+b), weighted by the parameter, *r*.

If exponential decay is slow, then a boost from a large input takes a long time to decay away. If the decay is fast, then previous boosts from input rapidly decay, and the process more closely tracks recent fluctuations. Thus, relatively faster exponential decay, with a>b, associates with closer tracking and pass through of signal fluctuations at higher frequencies.

### 5.2. Combining Low Pass Filters

Candia et al.’s model in Figure 2b combines the relatively fast, *F*, and slow, *S*, low pass filters shown in Figure 3a. The additive parallel model in Figure 2c combines the same two low pass filters in a different way. Both combinations yield the same overall system dynamics, shown as the blue curve in Figure 3a.

These models can be distinguished by reducing or blocking *F*. In Candia et al.’s model, reducing *F* will proportionally reduce the total output. In the additive parallel model, reducing *F* will not alter the slower, long term decay dynamics of *S*. Such a test would probably require a controlled experiment or an unusual situation that blocks one process but not the other.

The Laplace frequency analysis makes it easier to understand how component processes combine. In this case, the simple structure of these models would allow one to compare them without detailed frequency analysis. The next two models illustrate a more direct role for frequency analysis in comparing different processes.

### 5.3. High Pass Filters Ignore Slowly Changing Inputs

The model in Figure 2d processes input through a pair of sequential filters. One filter, *S*, passes low frequencies. This filter reduces the collective memory induced by high frequency, rapidly changing stimuli. Events that change rapidly often appear as background noise without a meaningful signal. Figure 3b shows this low pass filter in the green curve.

The other filter, H=G/S, passes high frequencies. This filter reduces the collective memory induced by low frequency, slowly changing stimuli. Events that change slowly may appear as a nearly constant background, which does not produce sufficient stimulus to register as a meaningful signal. Figure 3b shows this high pass filter in the gold curve.

The height of the curves is on a log scale. So multiplying the filters corresponds to adding the heights of the two curves. The sum yields the overall system response, *G*, in the blue curve. That curve is the same in all three panels.

If data could be obtained while bypassing one of the two processes, then the characteristics of the remaining component could be analyzed by measuring its frequency response.

### 5.4. Feedback and Resonant Frequencies

The model in Figure 2e has a primary fast exponential decay of memory by the process, *F*. The input into this feedback system is the difference between the current stimulus and the current memory, e=δ−m.

When the current stimulus is greater than the current memory, the self-correcting feedback system responds by increasing its memory. Similarly, when the current stimulus is less than the current memory, feedback reduces memory.

To match exactly to the system dynamics of the Candia et al. model, the feedback system filters the input difference signal by the process, *C*. The gold curve in Figure 3c shows that the filtering process has a resonant frequency peak. A resonant peak means that the filter enhances the input difference signal, *e*, when that signal has an intermediate frequency.

Enhancement of the difference input causes the self-correcting feedback system to reduce the difference more rapidly. In other words, the system moves its memory level more rapidly toward the current external stimulus level. The intermediate resonant peak causes the system to track more closely to external signals that are neither too slow nor too fast.

It may make sense for memory to track most closely to signals of intermediate frequency. Fast signals often arise from noisy fluctuations with little information. Slow signals set a background that can easily be tracked without rapid self-correcting adjustment.

Ideal tests would measure the response of the individual component processes or would block the feedback loop to measure the direct response of the CF pathway.

## 6. Conclusions

Candia et al.’s big data analysis focuses attention on a simple common pattern. That pattern sets the key challenge for understanding the mechanisms that influence collective memory.

In many fields of study, big data provide similar opportunities to characterize simple common patterns. Those patterns set the key challenge for the understanding of mechanistic process.

For the study of common patterns to mature, we require a stronger analytic approach to process. The first step should be consideration of the generic features of the pattern and the associated generic features that an explanatory model must have. The set of alternatives provides the basis for further empirical study.

I showed how a basic frequency response characterization of dynamics led immediately to four alternative models of process. By focusing on frequency, the models provide a potential approach to discriminate between the alternatives.

At present, there is no consistent and widely understood approach to forming a set of alternative explanations that can be tested empirically. I presented frequency analysis to illustrate how one method can help to form alternative explanations.

Big data will continue to characterize the commonly observed patterns of nature. Deriving insight from those patterns will require a broader understanding of how the commonly observed patterns arise. Better methods for generating and evaluating alternative mechanistic explanations will be crucial.
